
JOURNAL INFORMATION
==============================
NLM Title Abbreviation: Pregnancy (Hoboken)
Journal ID: Pregnancy (Hoboken)
NLM Journal ID: 9918994890806676
Journal ID: PMF2

Pregnancy

EISSN: 2997-9684
Publisher: Wiley

ARTICLE INFORMATION
==============================
PMCID: PMC13344800
PMID: 42597183
DOI: 10.1002/pmf2.12036
Article ID: PMF212036
Article version: 1
Subjects: Smfm 2025 Pregnancy Meeting, Conference Schedule

Conference Schedule

Electronic publication date: 2025 Jan 5
Publication date: 2025 Jan
Volume: 1
Issue: Suppl 1
Electronic Location ID: e12036
Copyright: © 2025 Society for Maternal‐Fetal Medicine. Pregnancy published by Wiley Periodicals LLC on behalf of the Society for Maternal‐Fetal Medicine.
License: This is an open access article under the terms of the http://creativecommons.org/licenses/by/4.0/ License, which permits use, distribution and reproduction in any medium, provided the original work is properly cited.
License URL: https://creativecommons.org/licenses/by/4.0/

Figure count: 0
Table count: 0
Page count: 2

source-schema-version-number: 2.0
cover-date: January 2025
details-of-publishers-convertor: Converter:WILEY_ML3GV2_TO_JATSPMC version:6.8.3 mode:remove_FC converted:07.07.2026

==============================

### Conference Schedule

### SMFM 2025 Pregnancy Meeting

SMFM2025 PREGNANCY MEETING™

Postgraduate Courses and Scientific and Clinical Meeting

Society for Maternal•Fetal Medicine

*January 27–February 1, 2025*
*Gaylord Rockies Resort and Convention Center*
*Aurora, Colorado*

**All abstract presentations take place at the Gaylord Rockies Resort and Convention Center.**

SCHEDULE OF ORAL PRESENTATIONS

### THURSDAY, January 30

**7:15 AM–8:00 AM** — **Opening Addresses and Awards**

**8:00 AM–10:00 AM** — **Oral Plenary Session 1**
*Abstracts 1-8 | Aurora Ballroom*

**10:00 AM–10:30 AM** — **Late-Breaking Research Presentations Session 1**
*Abstracts LB01-02 | Aurora Ballroom*

**10:30 AM–12:30 PM** — **Poster Session 1**
*Abstracts 105-384 | Exhibit Hall*

**1:30 PM–3:30 PM** — **Oral Concurrent Sessions**

Session 1 — **Equity, Public Health, and Policy**
*Abstracts 9-18 | Aurora Ballroom A*

Session 2 — **Clinical Obstetrics and Quality**
*Abstracts 19-28 | Aurora Ballroom B*

Session 3 — **Ultrasound and Genetics**
*Abstracts 29-38 | Aurora Ballroom CD*

**4:00 PM–6:00 PM** — **Poster Session 2**
*Abstracts 385-665 | Exhibit Hall*

### FRIDAY, January 31

**7:45 AM–8:00 AM** — **Foundation for SMFM Chair Address**

**8:00 AM–10:00 AM** — **Oral Plenary Session 2 – Fellows Plenary**
*Abstracts 39-46 | Aurora Ballroom*

**10:00 AM–10:30 AM** — **Late-Breaking Research Presentations Session 2**
*Abstracts LB03-04 | Aurora Ballroom*

**10:30 AM–12:30 PM** — **Poster Session 3**
*Abstracts 666-947 | Exhibit Hall*

**1:30 PM–3:30 PM** **Oral Concurrent Sessions**

**Session 4** **Basic and Translational Science**
*Abstracts 47-56* | *Aurora Ballroom A*

**Session 5** **Hypertension**
*Abstracts 57-66* | *Aurora Ballroom B*

**Session 6** **Prematurity and Newborn**
*Abstracts 67-76* | *Aurora Ballroom CD*

**4:30 PM–6:00 PM** **Poster Session 4**
*Abstracts 948-1227* | *Exhibit Hall*

### SATURDAY, February 1

**8:00 AM–10:15 AM** **Oral Concurrent Sessions**

**Session 7** **Diabetes**
*Abstracts 77-85* | *Aurora Ballroom A*

**Session 8** **Fetus and Fetal Intervention**
*Abstracts 86-94* | *Aurora Ballroom B*

**Session 9** **Medical Complications**
*Abstracts 95-104* | *Aurora Ballroom CD*

**10:15 AM–10:30 AM** **Late-Breaking Research Presentations Session 3**
*Abstract LB05* | *Aurora Ballroom A*

**10:15 AM–10:30 AM** **Late-Breaking Research Presentations Session 4**
*Abstract LB06* | *Aurora Ballroom B*

Topic Index
Author Index
Institution Index

Future Pregnancy Meeting Dates

SMFM 2026 Pregnancy Meeting™
February 8–13, 2026
Caesars Forum
Las Vegas, NV

SMFM 2027 Pregnancy Meeting™
March 21–26, 2027
Gaylord National Resort and Convention Center
National Harbor, MD

SMFM 2028 Pregnancy Meeting™
April 23–28, 2028
Caesars Forum
Las Vegas, NV
